# LOXHD1 is indispensable for coupling auditory mechanosensitive channels to the site of force transmission

**DOI:** 10.21203/rs.3.rs-3752492/v1

**Published:** 2024-01-02

**Authors:** Pei Wang, Katharine K. Miller, Enqi He, Siddhant S. Dhawan, Christopher L. Cunningham, Nicolas Grillet

**Affiliations:** 1 Department of Otolaryngology-Head and Neck Surgery, Stanford University, 240 Pasteur Drive, Stanford, CA, USA; 2 Pittsburgh Hearing Research Center, Department of Otolaryngology, University of Pittsburgh, Pittsburgh, PA 15213, USA; 3 Lead contact

## Abstract

Hearing is initiated in hair cells by the mechanical activation of ion channels in the hair bundle. The hair bundle is formed by stereocilia organized into rows of increasing heights interconnected by tip links, which convey sound-induced forces to stereocilia tips. The auditory mechanosensitive channels are complexes containing at least four protein-subunits – TMC1/2, TMIE, CIB2, and LHFPL5^1–16^ – and are located at the tips of shorter stereocilia at a yet-undetermined distance from the lower tip link insertion point^17^. While multiple auditory channel subunits appear to interact with the tip link, it remains unknown whether their combined interaction alone can resist the high-frequency mechanical stimulations owing to sound. Here we show that an unanticipated additional element, LOXHD1, is indispensable for maintaining the TMC1 pore-forming channel subunits coupled to the tip link. We demonstrate that LOXHD1 is a unique element of the auditory mechanotransduction complex that selectively affects the localization of TMC1, but not its close developmental paralogue TMC2. Taking advantage of our novel immunogold scanning electron microscopy method for submembranous epitopes (SUB-immunogold-SEM), we demonstrate that TMC1 normally concentrates within 100-nm of the tip link insertion point. In LOXHD1’s absence, TMC1 is instead mislocalized away from this force transmission site. Supporting this finding, we found that LOXHD1 interacts selectively *in vitro* with TMC1 but not with TMC2 while also binding to channel subunits CIB2 and LHFPL5 and tip-link protein PCDH15. SUB-immunogold-SEM additionally demonstrates that LOXHD1 and TMC1 are physically connected to the lower tip-link complex *in situ*. Our results show that the TMC1-driven mature channels require LOXHD1 to stay coupled to the tip link and remain functional, but the TMC2-driven developmental channels do not. As both tip links and TMC1 remain present in hair bundles lacking LOXHD1, it opens the possibility to reconnect them and restore hearing for this form of genetic deafness.

## Introduction

**Figure 1:**
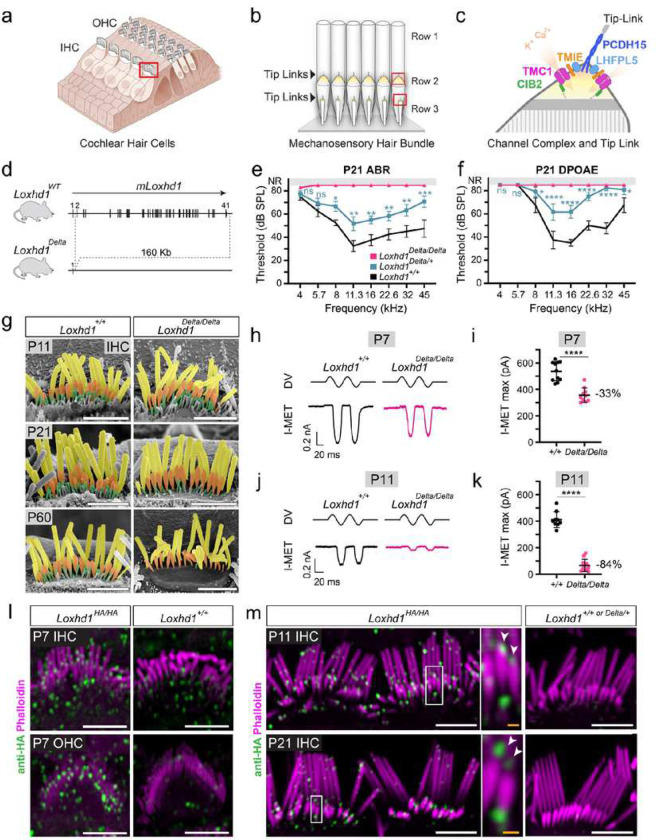
The absence of LOXHD1 leads to hair bundle mechanotransduction deficits and localizes at transducing stereocilia row 2 tips. **a**, Illustration of a cochlear sensory epithelium, which contains IHCs and OHCs that sense sound-induced forces with their apical hair bundles. **b**, Illustration of a P21 IHC hair bundle comprising three stereocilia rows interconnected by tip links. **c**, Cartoon representing the known auditory MET channel complex subunits located near the lower tip-link insertion point. **d**, The *Loxhd1*^*Delta*^ allele contains a large-scale genomic deletion at the *Loxhd1* locus (see also Extended Data Fig.1). **e**, ABR and **f**, DPOAE thresholds of P21 *Loxhd1*^*Delta*^ mice across a 4–45 kHz frequency range. Data are represented as mean ± SEM (*Loxhd1*^+/+^, n = 4; *Loxhd1*^*Delta/+*^, n = 6; *Loxhd1*^*Delta/Delta*^, n = 5. Two-way ANOVA (frequency/genotype) followed by the Dunnett multiple comparisons test, with WT means being the reference. *Loxhd1*^*Delta/Delta*^ mice values are significantly different from *Loxhd1*^+/+^. For all tests in this paper, ns = P > 0.05; * = P≤ 0.05; ** = P ≤ 0.01; *** = P ≤ 0.001; **** = P ≤ 0.0001. *Loxhd1*^*Delta/Delta*^ thresholds are significantly different from *Loxhd1*^+/+^ between 5.7–45 kHz for ABR and 8–45 kHz for DPOAE (see Extended Data Table 1 for group numbers and P values. Only comparisons between *Loxhd1*^+/+^ and *Loxhd1*^*Delta/+*^ are indicated on the graph. **g**, SEM of apical IHC *Loxhd1*^*Delta*^ mice at P11, P21, and P60, with each stereocilia row being colored in post-production. Scale bars = 3 μm. **h-k**, Mechanotransduction current (I-MET) traces from P7 (h) and P11 (j) IHC *Loxhd1*^+/+^ and *Loxhd1*^*Delta/Delta*^ mechanically stimulated with a fluid jet and the maximum I-MET currents recorded (i and k). P7: *Loxhd1*^+/+^: n_mice_ = 7, n_cells_ = 11; *Loxhd1*^*Delta/Delta*^ n_mice_ = 10, n_cells_ = 10; P11: *Loxhd1*^+/+^: n_mice_ = 6, n_cells_ = 9; *Loxhd1Delta/Delta* n_mice_ = 9, n_cells_ = 11. Unpaired two-tailed t test. DV = driving voltage of the fluid jet piezoelectric stimulator. **l-m**, Hair bundle fluorescent labeling the actin-rich stereocilia, and with an anti-HA antibody to detect LOXHD1 in *Loxhd1*^*HA/HA*^ animals. Specific HA staining is detected in *Loxhd1*^*HA/HA*^ apical IHC and OHC HBs at P7 (l), P11 and P21 (m) in but not in negative controls without HA. Scale bars = 4 μm. LOXHD1-HA can clearly be detected at the tip of row 2 stereocilia (arrowheads in insets) of apical P11 and P21 IHCs. White scale bars = 4 μm, orange scale bars = 0.4 μm.

Our senses of hearing and touch rely on mechanotransduction (MET), the conversion of mechanical forces into electrical currents. Molecular solutions to MET are radically different between these senses: touch relies mainly on a single molecule forming a mechanosensitive ion channel, PIEZO2^18,19^. Contrarily, hearing requires the assembly of at least four distinct proteins to form a functional ion channel complex: TMC1/TMC2, TMIE, CIB2, and LHFPL5^1–16^. To sense sound-induced forces, MET proteins must be transported, positioned, and maintained at precise sites within the hair bundle (HB). The HB is an apical membrane specialization of cochlear hair cells comprised of an array of actin-filled protrusions called stereocilia arranged in rows of increasing heights and displaced by sound-induced forces (Fig.1a–b)^20,21^. Stereocilia from different rows are interconnected by tip links comprising PCDH15 and CDH23, which convey displacement forces to the extreme tip of smaller stereocilia^22–27^. It is at this site, at the membrane of shorter stereocilia tips, that auditory ion channels localize and are mechanically gated by the tip link^17,28^ (Fig. 1c).

The mechanosensitive channel comprises pore-forming subunits TMC1 or TMC2, along with TMIE^3,4,11^. In rodents, TMC1 and TMC2 are dynamically expressed in cochlear hair cells during development, with TMC2 first present around birth, followed by a short period in which TMC2 and TMC1 are coexpressed (from postnatal day (P)3) and ending with the maintenance of TMC1 alone in mature cochlear hair cells occurring at P7 for outer hair cells (OHC) and at P11 for inner hair cells (IHCs)^1,11,16,29^. In contrast to these dynamic expression patterns, TMIE, CIB2, and LHFPL5 are expressed at the earliest stages at which MET currents can be elicited ^5,7,9,10,13,30^. Each channel complex protein interacts with multiple other subunits, and many complex proteins depend on other subunits for proper localization^5,8–13,31–37^. For instance, in *Tmie*^*KO*^ and *Cib2*^*KO*^ hair cells, TMC1 and TMC2 do not localize to the HB^8,11,31^, a phenotype that also progressively develops in *Lhfpl5*^*KO* 12^.

The existence of a direct, physical connection between the channel complex and the tip link is supported by two lines of results: First, TMC1/2, TMIE, and LHFPL5 can all bind *in vitro* to PCDH15, which constitutes the lower part of the tip link^10–13,33,35^. Second, from dominant negative experiments in zebrafish stereocilia: endogenous PCDH15 is redistributed broadly after the overexpression of membrane-bound TMC2a-N-terminal fragments in zebrafish^33^. However, *in situ* confirmation through high-resolution localization of the channel subunits at the site of tip link insertion at the stereocilia tips is still lacking^29,34,38^. Finally, while the biochemical data involving multiple interactions suggest a strong connection between the channel complex to the tip link, it is unknown whether their combination is sufficient to maintain the coupling *in vivo* during high-frequency mechanical stimulation owing to sounds.

Here, we demonstrate that LOXHD1 is a novel MET complex component, critical to preserve the cohesiveness and coupling of the channel complex and the tip link.

## Results

### A large-scale genomic deletion of *Loxhd1* in mice leads to semi-dominant hearing loss

Mutations in the gene *LOXHD1* cause recessive hearing loss in Humans (DFNB77), mouse and dogs^39–43^ but is molecular function remains elusive. LOXHD1 is composed of up to 15 PLAT (polycystin lipoxygenase alpha-toxin) repeats and one coiled-coil domain^39^. To comprehensively characterize the function of the deafness gene *Loxhd1*’s in hair cells and prevent functional compensation from alternative splicing isoforms, we used CRISPR/Cas9 genomic editing in mouse zygotes to delete 160 kb of the *Loxhd1* locus downstream of the first exon and inactivate all 40 exons encoding the 15 PLAT repeats. (Fig.1d and Extended Data Fig. 1). The deleted interval did not contain other protein-coding genes. We named this allele *Loxhd1*^*Delta*^. The *Loxhd1*^*Delta/Delta*^ mice were viable and showed no sign of abnormal behavior at least until P30. Additionally, we evaluated the hearing of *Loxhd1*^*Delta*^ littermates at P21. The *Loxhd1*^*Delta/Delta*^ animals were profoundly deaf, showing no auditory brain stem responses (ABR) at an 80-dB sound pressure level (SPL) across frequencies except for some residual responses at 4 kHz (Fig. 1e). The hearing of the heterozygous *Loxhd1*^*Delta*/+^ animals was also affected, which had ABR thresholds elevated at all frequencies from 8 to 45 kHz compared to wild-type (WT) littermates. Because this degree of hearing loss is compatible with an OHC dysfunction, we measured the distortion products of otoacoustic emission (DPOAE) of the P21 *Loxhd1*^*Delta*^ animals. DPOAE thresholds were not detected at an 80-dB SPL across frequencies in *Loxhd1*^*Delta/Delta*^ and were elevated at all frequencies starting at 8 kHz in *Loxhd1*^*Delta/+*^ compared with their WT littermates (Fig. 1f). Therefore, LOXHD1 protein(s) is required for cochlear hair cell function, and appropriate levels are important for optimal function because even partial decreases (i.e., in *Loxhd1*^*Delta/+*^*)* have significant impacts. Thus, the dosage and the nature of LOXHD1 splicing isoforms are crucial factors for hair cell function and hearing. Although ABRs were not detected at P21, IHCs and OHCs presented with only mild HB morphological defects: HBs of *Loxhd1*^*Delta/Delta*^ apical IHCs showed a late thinning and progressive disappearance of row 3 stereocilia at P21, while row 2 tip shape and stereocilia width were severely affected only at P60 (Fig. 1g and Extended Data Fig. 2a-g). Notably, tip links were still detected in P21 *Loxhd1*^*Delta/Delta*^ IHCs (Extended Data Fig. 2h). HBs of *Loxhd1*^*Delta/Delta*^ apical OHCs showed increased variability in height in row 3 stereocilia heights at P11 (Extended Data Fig. 2i-k, Extended Data Table 1). Overall, hair cell dysfunction caused by the absence of LOXHD1 appears not to result directly from HB morphological defects but more likely stems from MET defects.

### The absence of LOXHD1 induces a progressive IHC MET current reduction at P7 and P11

We previously showed that when LOXHD1 was mutated at the PLAT10 repeat, the MET current amplitudes of homozygous mutant IHCs at P7 were comparable with controls^44^. However, at P11, the amplitudes were subsequently reduced by 95%^44^. In the *Loxhd1*^*Delta/Delta*^ animals at the same ages, we measured MET currents using whole-cell patch-clamp generated by fluid jet mechanical stimulation. The deflection of P7 IHC HBs toward the tallest stereocilia row of *Loxhd1*^*+/+*^ and *Loxhd1*^*Delta/Delta*^ produced inward currents (Fig. 1h). However, while the maximum MET current (I-MET) was 537 ± 73 pA (SD) in *Loxhd1*^*+/+*^, it was significantly reduced to 357 ± 54 pA in *Loxhd1*^*Delta/Delta*^ (Fig. 1i). At P11, the IHC I-MET currents were even further reduced from 412 pA ± 60 pA in *Loxhd1*^*+/+*^ to 66 pA ± 46 pA in *Loxhd1*^*Delta/Delta*^ littermates (Fig. 1j–k). Therefore, in contrast to our previous work with localized mutations of *Loxhd1*, the complete absence of LOXHD1 leads to earlier effects on IHC MET currents at P7 (33% amplitude reduction), which continues to an 84% reduction at P11. In conclusion, the analysis of the *Loxhd1*^*Delta*^ allele has demonstrated that LOXHD1 is required for IHC MET at earlier stages (P7), while it was not required at this time point in the previous PLAT10 *Loxhd1* mutants^44^. Thus, we conclude that functional compensation by *Loxhd1* splicing isoforms occurred in mouse mutations in which LOXHD1 is mutated only in PLAT10. Furthermore, as small MET-currents (~<100 pA) are still detected at P11, it appears that some MET channels can still function to a limited degree in matured IHCs when LOXHD1 is absent.

### LOXHD1 localizes to the HB and is enriched at the tips of transducing stereocilia

To understand the molecular mechanism by which LOXHD1 affects MET channel function, we determined its localization. We generated a tagged knock-in (KI) mouse, with the insertion of an HA-sequence in the first *Loxhd1* exon before the first PLAT repeat (Extended Data Fig. 3a and Methods). Homozygous animals for the *Loxhd1*^*HA*^ allele showed normal ABR and DPOAE responses at P30, demonstrating that the epitope tag insertion did not affect LOXHD1 and hair cell function (Extended Data Fig. 3a-b). We determined the LOXHD1 expression pattern in cochlear HBs from P7 to P21 by immunofluorescence staining against the HA tag and fluorescent-phalloidin to label stereocilia, comparing the results from *Loxhd1*^*HA/HA*^ and non-HA animals. At P7, specific LOXHD1-HA signal was present in HB of IHC but also of OHC where it appeared to delineate two stereocilia heights, likely corresponding to the tips of row 2 and 3 stereocilia (Fig. 1l). In P11 and P21 IHC HBs, which have tall stereocilia rows allowing fine localization, the LOXHD1-HA signal was enriched at the tip of row 2 stereocilia and potentially also at the tip of row 3 stereocilia, which are weakly stained by phalloidin (Fig. 1m). The LOXHD1-HA staining was also found to a lesser degree along the height of row 1 stereocilia but not with the expected pattern of the upper tip-link insertion sites. The LOXHD1 staining was specific because it was absent in the non-HA control HBs. We infer from these results that LOXHD1 proteins localize preferentially at the tip of row 2 and potentially row 3, colocalizing in the same subcellular domain in which the MET channel complexes are situated and where the lower part of the tip link inserts into the stereocilia membrane.

### The absence of LOXHD1 reduces the number of IHC row 2 stereocilia with TMC1 at their tip

**Figure 2:**
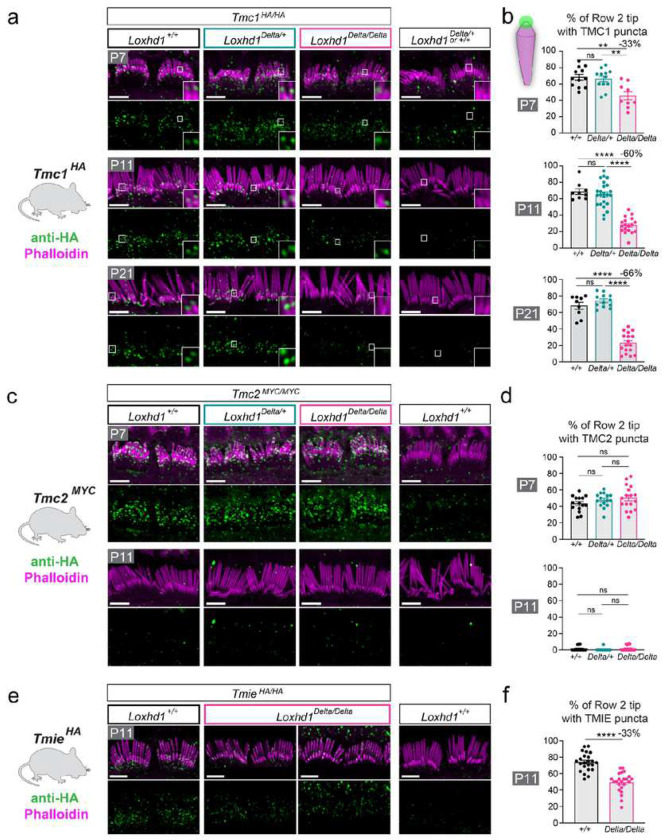
TMC1 is progressively missing from row 2 stereocilia tips of IHCs in the absence of LOXHD1. **a**, TMC1 immunofluorescence localization (anti-HA on *Tmc1*^*HA/HA*^ animals) at the tips of row 2 IHC stereocilia at P7, P11, and P21 showed a progressive reduction in *Loxhd1*^*Delta/Delta*^ but not in *Loxhd1*^*Delta/+*^. Insets show high magnification at some row 2 tips. Scale bars = 4 μm. **b**, Percentage of row 2 tips with TMC1 puncta identified from 3D-volumes per genotype (see Extended Data Fig. 2 and the Material and Methods section for more details). Per group: n_mice_ ≥ 3, n_cells_ ≥ 9. One-way ANOVA followed by the Tukey multiple comparisons test (see Extended Data Table 1 for group numbers and P values for all panels). **c**, TMC2 immunofluorescence localization (anti-MYC on *Tmc2*^*MYC/MYC*^ animals) at the tips of row 2 IHC stereocilia at P7 and P11. Scale bars = 4 μm. **d**, The percentage of row 2 tips with TMC2 puncta was not affected in *Loxhd1*^*Delta/Delta*^ at P7 and P11. Per group: n_mice_ ≥ 3, n_cells_ ≥ 9. One-way ANOVA followed by the Tukey multiple comparisons test. **e**, TMIE immunofluorescence localization (anti-HA on *Tmie*^*HA/HA*^ animals) at the tips of row 2 IHC stereocilia at P11. Scale bars = 4 μm. **f**, The percentage of row 2 tips with TMIE puncta was reduced in *Loxhd1*^*Delta/Delta*^ at P11 but to a lesser extent than TMC1. Per group: n_mice_ ≥ 3, n_cells_ ≥ 9. Unpaired two-tailed t test.

**Figure 3:**
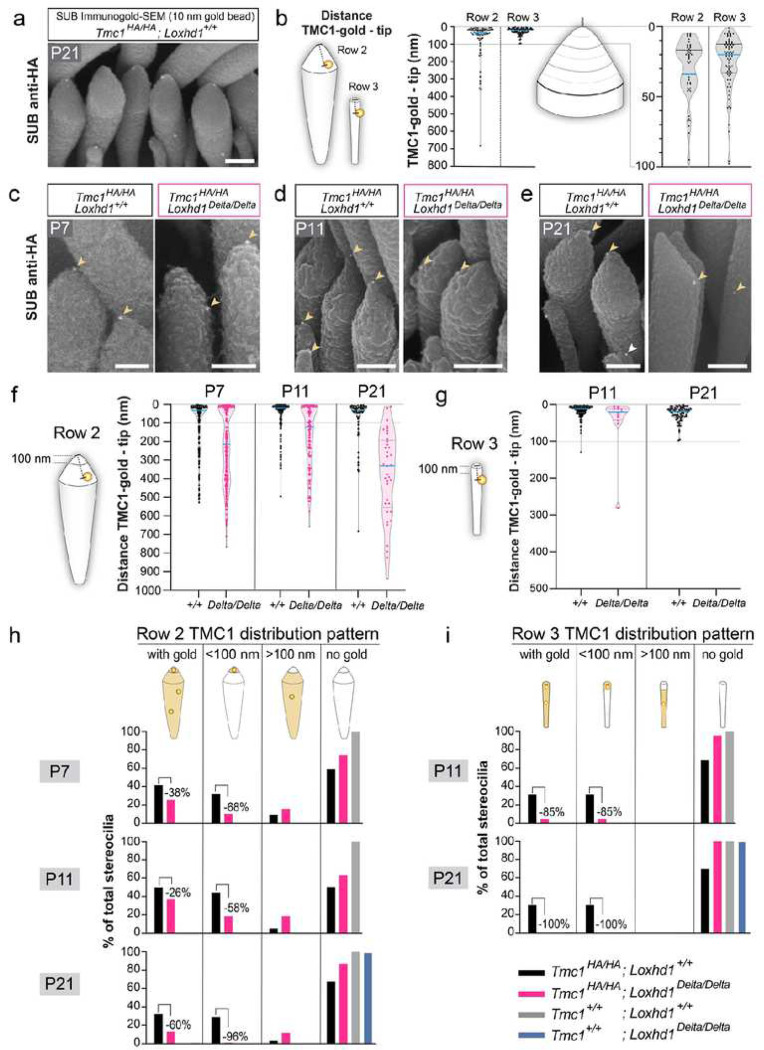
TMC1 is progressively mislocalized away from the first 100 nm of row 2 stereocilia tips of IHCs in the absence of LOXHD1. **a**, Sub-membranous (or SUB) immunogold-SEM anti-HA with secondary antibody conjugated to 10 nm gold-beads localizes TMC1 at the tip of rows 2 and 3 stereocilia of P21 IHCs. Note that some row 3 stereocilia are disconnected from row 2, while gold-beads are attached to row 2 shafts at the presumptive upper tip link insertion position. Scale bar = 200 nm. **b,** Distance between TMC1-gold beads to the stereocilia tip in rows 2 and 3 stereocilia measured from SUB-immunogold-SEM micrographs in *Loxhd1*^+/+^ IHCs. TMC1-gold beads are concentrated within 100 nm from the stereocilia tips (see the focused panel). Medians are indicated by a blue line. P21: n_mice_ = 1; n_cells_ = 13; row 2: n_stereocilia_ = 40; n_gold_ = 62; row 3: n_stereocilia_ = 45; n_gold_ = 77. **c-e,** SUB-immunogold-SEM anti-HA detects TMC1-associated gold beads (arrowheads) at IHC row 2 tips of *Loxhd1*^+/+^ and *Loxhd1*^*Delta/Delta*^ at P7, P11, and P21. Scale bar = 200 nm. **f,** Distance between TMC1-gold beads to the stereocilia tip in row 2 stereocilia measured from SUB-immunogold-SEM micrographs in *Loxhd1*^+/+^ and *Loxhd1*^*Delta/Delta*^ IHCs. Per group: P7: n_mice_ ≥ 2; n_cells_ ≥ 18; n_stereocilia_ ≥ 113; n_gold_ =168 for *Loxhd1*^+/+^ and 194 for *Loxhd1Delta/Delta*; P11: n_mice_ = 2; n_cells_ ≥ 16; n_stereocilia_ ≥ 51; n_gold_ ≥ 62; n_gold_ 204 for *Loxhd1*^+/+^ and 62 for *Loxhd1*^*Delta/Delta*^; P21: n_mice_ ≥ 1; n_cells_ ≥ 13; n_stereocilia_ ≥ 30; n_gold_ = 62 for *Loxhd1+/+* and 37 for *Loxhd1Delta/Delta* (see Extended Data Table 1). Medians are indicated by a blue line. **g,** Distance between TMC1-gold beads to the stereocilia tip in row 3 stereocilia measured from SUB-immunogold-SEM micrographs in *Loxhd1*^+/+^ and *Loxhd1*^*Delta/Delta*^ IHCs. Per group: P11: n_mice_ = 2; n_cells_ ≥ 14; n_stereocilia_ = 117 for *Loxhd1*^+/+^ and 12 for *Loxhd1*^*Delta/Delta*^; n_gold_ = 135 for *Loxhd1*^+/+^ and 62 for *Loxhd1*^*Delta/Delta*^; per group: P21: n_mice_ ≥ 1; n_cells_ ≥ 13; n_stereocilia_ = 45 for *Loxhd1*^+/+^ and 0 for *Loxhd1*^*Delta/Delta*^; ngold = 62 for *Loxhd1*^+/+^ and 0 for *Loxhd1*^*Delta/Delta*^ (see Extended Data Table 1). **h-i**, Stereocilia classification based on the TMC1-gold distribution pattern (present in the stereocilia, with at least one gold bead in the first 100 nm, with at least one gold bead below 100 nm, and without any gold beads). Row 2 (h): per group: P7: n_mice_ ≥ 1; n_cells_ ≥ 16; n_stereocilia_ ≥ 166; P11-P21: n_mice_ ≥ 1; n_cells_ ≥ 13; n_stereocilia_ ≥ 130; row 3 (i): per group: P11: n_mice_ ≥ 1; n_cells_ ≥ 13; n_stereocilia_ ≥ 148; P21: n_mice_ = 1; n_cells_ ≥ 11; n_stereocilia_ ≥ 65 (see Extended Data Table 1).

Because the absence of LOXHD1 affects IHC MET current progressively between P7 and P11 while HB morphology remained normal, we hypothesized that one or more components of the mature MET channel complex are selectively affected. The only known channel complex subunit required for mature HB MET but not for MET during development is TMC1^14^. IHCs first express the paralogue TMC2 from P1–P2, which can compensate for TMC1 deficiency until P7–P10 when its expression fades away ^1,16,29,45^. To test if the lack of LOXHD1 protein would affect TMC1 HB localization, we tracked TMC1 endogenous protein using the *Tmc1*^*HA/HA*^ KI mice, which we bred with the *Loxhd1*^*Delta*^ mice^11^. We performed anti-HA immunofluorescence staining on apical IHC and imaged HB with high-resolution Airyscan to detect TMC1 at P7, P11, and P21 in littermates and used non-HA animals as negative controls. In the control animals (*Tmc1*^*HA/HA*^; *Loxhd1*^*+/+*^), TMC1-HA was detected at low levels in the HB and formed puncta mainly at the tip of row 2 stereocilia at all ages tested (Fig. 2a). In hair cells lacking LOXHD1 (*Tmc1*^*HA/HA*^; *Loxhd1*^*Delta/Delta*^) at P7, TMC1-HA was present in the HB and appeared also as puncta at the tip of row 2 stereocilia. However, the number of puncta seemed reduced compared with the controls (*Tmc1*^*HA/HA*^; *Loxhd1*^*+/+*^*)*. To quantify the number of row 2 stereocilia with a punctum at their tip, we used a 3D-surface rendering of the actin-core (labeled with phalloidin) and of the TMC1-HA staining (Extended Data Fig. 4a, Extended Data Movie 1, and Methods). In hair cells from the P7 *Loxhd1*^*+/+*^ mice, 68% ± 12% (SD) of stereocilia per HB had a TMC1 punctum. Relative numbers were similar for *Loxhd1*^*Delta/+*^, while for *Loxhd1*^*Delta/Delta*^, the proportion of row 2 tips with a TMC1 punctum was significantly reduced to 46% ± 15% (Fig. 2b). As hair cells matured, the proportion of row 2 stereocilia tips with a TMC1 punctum was further reduced to 28 ± 10% per HB at P11 and 23% ± 12% per HB at P21 (Fig. 3a–b). Normalized to the WT values, the proportion of TMC1-positive row 2 stereocilia tips decreased by 33% at P7, by 60% at P11, and by 66% at P21. These results indicate that in IHCs, the absence of LOXHD1 leads to a progressive reduction of the proportion of row 2 stereocilia with TMC1 puncta, a molecular phenotype that is consistent with the progressive reduction in MET current amplitudes. Importantly, IHC from heterozygous *Loxhd1*^*Delta/+*^ were not affected by this phenotype.

### The absence of LOXHD1 does not affect TMC2 or LHFPL5 localization in the HB but partially affects TMIE

To determine if the absence of LOXHD1 would affect the additional subunits of the MET channel complex, we localized other known subunits to the tips of stereocilia in the HBs using the tagged-KI mice or validated antibodies. First, we inspected TMC2 expression using the *Tmc2*^*MY*C^ allele^11^. At P7, TMC2 was found in the HB and at the tip of row 2 apical IHC stereocilia in the controls (*Tmc2*^*MYC/MYC*^; *Loxhd1*^*+/+*^) and had virtually disappeared by P11 (Fig. 2c). The proportion of row 2 stereocilia with TMC2-tip puncta was similar between the *Loxhd1*^*+/+*^ *and Loxhd1*^*Delta/Delta*^ animals at P7 (43% ± 9% and 50% ±14%) and at P11 when TMC2 expression faded away (1.5% ± 3% and 1.8% ± 3%) (Fig. 2d). Therefore, the absence of LOXHD1 has no effect on TMC2 HB row 2 tip localization or expression time.

Subsequently, we evaluated TMIE, a subunit contributing to the channel pore, which is required for localization of TMC1 and TMC2 to the HB^11,31^. To localize TMIE, we used the *Tmie*^*HA*^ KI allele^11^. At P11, in the apical IHCs of the *Tmie*^*HA/HA*^; *Loxhd1*^*+/+*^ control mice, TMIE was found in 74% ± 11% of row 2 tips, while it was reduced to 49% ± 12% in apical IHCs of the *Tmie*^*HA/HA*^; *Loxhd1*^*Delta/Delta*^ mice (Fig. 2e–f). Therefore, the absence of LOXHD1 partially reduces the proportion of row 2 tips with TMIE puncta by 33% at P11.

We then looked at the protein CIB2, an auxiliary subunit required for TMC1 and TMC2 localization to the HB^8^. We used a commercially available antibody validated on *Cib2*^*KO*^ HBs^5,9^. As in these reports, we obtained a signal along the length of the stereocilia and a weak signal at the tip of row 2 stereocilia in IHCs from the P11 *Loxhd1*^*+/+*^ mice (Extended Data Fig. 4b). As this signal was still present in the *Loxhd1*^*Delta/Delta*^ littermates, it appears that the absence of LOXHD1 does not change CIB2 HB localization.

Finally, we inspected LHFPL5, which is required for the proper numbers of tip links and TMC1 localization to the HB^12,13^. LHFPL5 was localized with an antibody previously validated on *Lhfpl5*^*KO*^ cochlear tissue^38^. LHFPL5 was found at the tips of row 2 stereocilia in a similar proportion between *Loxhd1*^*+/+*^ (81% ±11%) and *Loxhd1*^*Delta/Delta*^ (78% ±10%) in P11 apical IHCs (Extended Data Fig. 4c-d). These results indicate that LHFPL5 localization was not affected by LOXHD1 absence.

Overall, the absence of LOXHD1 had a selective effect on TMC1 row 2 tip localization as three other known MET machinery components – TMC2, CIB2, and LHFPL5 – appeared unaffected and as TMIE was only partially affected.

### TMC1 localizes within 100 nm from the stereocilia tips, where the tip links insert

To gain a more precise understanding of the distribution of TMC1 pore-channel subunits at the stereocilia tips, we used electron microscopy (EM). Instead of using immunogold-transmission EM (TEM) or immunogold focused-ion beam scanning EM (FIB-SEM), which are cumbersome and restricted to only a small sample number, we improved the existing immunogold-SEM protocol, previously limited to external epitopes^46–49^. We optimized the permeabilization, membrane preservation, antibody penetration, and post-fixation onto the tissue to resist the harsh steps of SEM sample preparation. With this method, named SUB-immunogold-SEM (Wang et al., *submitted*), we can now detect submembranous epitopes using a secondary antibody conjugated to a gold bead, which will generate backscattered electrons at the cell surface when imaged by SEM. SUB-immunogold-SEM allows nanoscale measurements and quantifications from large sample numbers. We performed anti-HA SUB-immunogold-SEM on control P21 IHCs (*Tmc1*^*HA/HA*^; *Loxhd1*^*+/+*^) using a 10-nm gold bead conjugated secondary antibody. We detected TMC1 gold beads at the tip of row 2 and the tip of row 3 stereocilia at P21, while none were detected in non-HA animals (Fig. 3a and Extended Data Fig. 5a). We measured the distance from the tips of the stereocilia to the center of the gold beads. The TMC1 gold beads were concentrated within 100 nm from the stereocilia tips in row 2 (81% of all gold beads) and row 3 (100%) (Fig. 3b). Within the first 100 nm, the TMC1 gold beads were further enriched within 50 nm from the tips of row 2 (73%) and row 3 stereocilia (90%) (Fig. 3b). Taken together, we demonstrate that in the IHCs of juvenile mice, the TMC1 pore-forming subunits of the auditory MET channels are clustered at the pointiest part of stereocilia tips, where the lower part of the tip link inserts. Further, despite their differences in shape and width of their tips, we found that rows 2 and 3 P21 IHC stereocilia present similar TMC1 distribution.

### TMC1 is progressively mislocalized from the stereocilia tips of IHCs in the absence of LOXHD1

In addition to the reduction of TMC1 puncta at the tips of row 2 stereocilia found in LOXHD1’s absence, we noticed by immunofluorescence that some remaining TMC1 puncta were frequently detected below the row 2 tip at P21 (Extended Data Fig. 5b). However, fluorescence microscopy was unable to distinguish whether this TMC1 signal indicated a mislocalization within row 2 stereocilia or if it was located at the tips of row 3 stereocilia, which is only weakly stained by phalloidin. Therefore, we repeated anti-HA SUB-immunogold-SEM experiments on IHCs from the *Loxhd1*^*+/+*^ and *Loxhd1*^*Delta/Delta*^ littermates at P7, P11, and P21 (Fig. 3c–e). We measured the distance from the tip of row 2 stereocilia to the center of the gold beads. In control hair cells (*Loxhd1*^*+/+*^), the TMC1 gold beads were increasingly concentrated in the first 100 nm of the row 2 tips with age (69% at P7, 85% at P11, and 81% at P21). In *Loxhd1*^*Delta/Delta*^ hair cells, the TMC1 gold beads were far less concentrated in the first 100 nm from row 2 tips (38% at P7, 44% at P11, and 8% at P21) and were progressively found at a longer distance from the stereocilia tips, culminating at P21 when the beads were distributed broadly all along the length of the stereocilia (Fig. 3f). We also measured the distance from the tip of row 3 stereocilia to the TMC1 gold beads at P11 and P21 in WT and found an even stronger concentration in the first 100 nm (99% at P11 and 100% at P21) (Fig. 3g). On a *Loxhd1*^*Delta/Delta*^ background, the TMC1 gold beads were hardly found in row 3 stereocilia, and the few found at P11 were mainly within the first 100 nm.

To determine how this altered TMC1 distribution affected the number of activatable stereocilia, we classified them according to the presence or absence of the TMC1 gold beads either at any distance from the tip or within the first 100 nm. The number of row 2 stereocilia with gold at any position decreased in the absence of LOXHD1 by 38% at P7, by 26% at P11, and by 60% at P21 (Fig. 3h). The proportion of stereocilia with the TMC1 gold beads in the first 100 nm decreased even more by 68% at P7, by 58% at P11, and by 96% at P21. At the same time, the proportion of stereocilia with gold lower than 100 nm from the tip increased, consistent with TMC1 mislocalization. When performed on row 3, this classification analysis showed a massive reduction of stereocilia with the TMC1 gold beads (85% decrease at P11 and 100% decrease at P21), with the remaining TMC1 beads localizing in the first 100 nm (Fig. 3i). Critically, in hair cells from our negative control animals without the *Tmc1*^*HA*^ allele (*Tmc1*^*+/+*^), either *Loxhd1*^*+/+*^ or *Loxhd1*^*Delta/Delta*^, virtually no gold was found in stereocilia in any experiments (Fig. 3h–i).

Overall, these SUB-immunogold-SEM experiments support that LOXHD1 is required for the proper expression, targeting, and/or maintenance of TMC1 in IHC stereocilia, similar to other MET-associated proteins. Importantly, LOXHD1 is the first protein identified that is critical for the localization of TMC1 within the first 100 nm of the row 2 tips, where the tip links insert and where the mechanical stimuli are received.

### The absence of LOXHD1 leads to TMC1 mislocalization in OHCs

As LOXHD1 and TMC1 were co-expressed in OHCs, we tested if TMC1 HB localization in OHCs was also affected by the *Loxhd1*^*Delta*^ allele, focusing on P7. We measured TMC1 fluorescence intensity in P7 OHC whole HB at apical, medial, and basal positions using the *Tmc1*^*HA*^ allele (Extended Data Fig. 6a). In WT, basal OHC HBs showed 2.3 times more signal than the apical ones (Extended Data Fig. 6b). In the *Loxhd1*^*Delta/Delta*^ HBs, the TMC1 signal was reduced at all tonotopic positions and more severely at the base (−61%) still maintaining a higher TMC1 expression than at the apex (1.5 times). Importantly, the TMC1 HB signal was also found to be reduced in heterozygous *Loxhd1*^*Delta/+*^ basal OHCs (−21%) compared with the WT littermates (Extended Data Fig. 6b). To assess how *Loxhd1* alleles affected TMC1 stereocilia localization in OHCs, we used SUB-immunogold-SEM on OHCs (Extended Data Fig. 6c). In WT, the TMC1 gold beads were enriched in the first 100 nm from row 2 and 3 tips (73% and 97% of all beads, respectively) (Extended Data Fig. 6d). In the *Loxhd1*^*Delta/Delta*^ littermates, TMC1 was comparably enriched at row 2 tips (66%) but reduced at row 3 tips (62%) (Extended Data Fig. 6d). In the absence of LOXHD1, the number of stereocilia with TMC1 was reduced in row 2 (−54%) and more drastically in row 3 (−71%), which also shows TMC1 mislocalization from the first 100 nm (Extended Data Fig. 6e-f). Therefore, as for IHCs, OHCs without LOXHD1 protein also exhibit TMC1 mislocalization in transducing stereocilia at P7.

### The absence of LOXHD1 leads to BAIAP2L2 mislocalization

**Figure 4:**
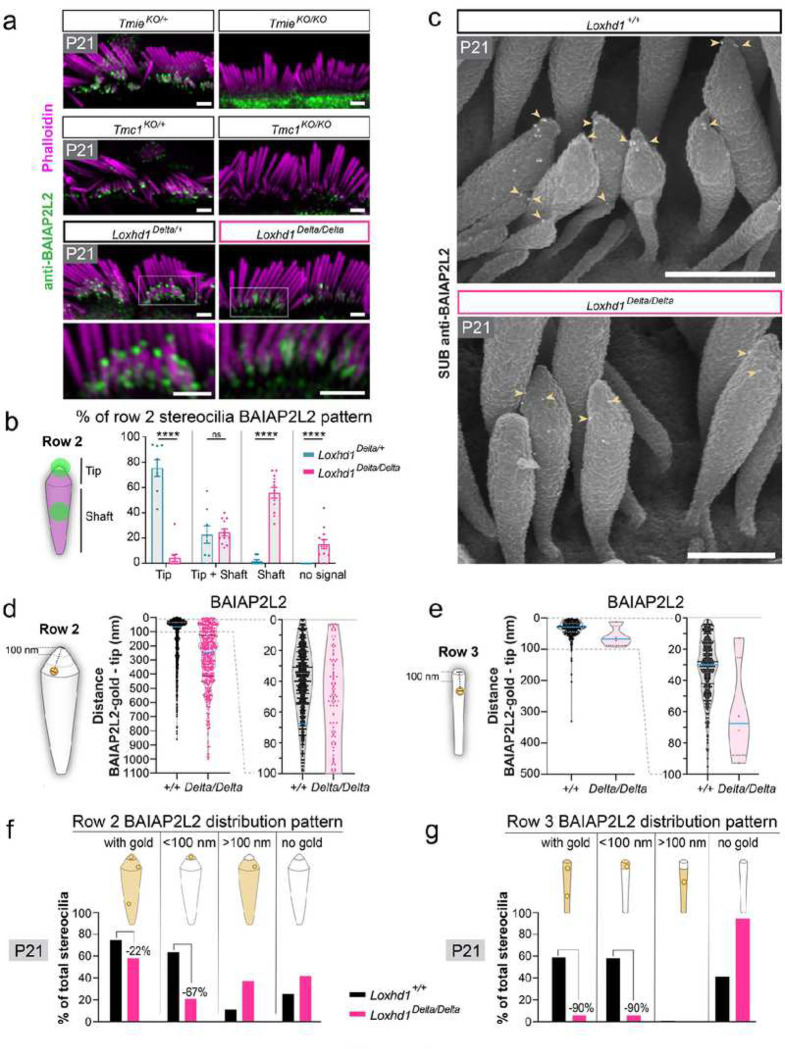
Stereocilia Ca^2+^ entry zones, probed with BAIAP2L2, are relocated from tips to shaft. **a,** Anti-BAIAP2L2 immunofluorescence on P21 apical HCs shows a strong signal at transducing stereocilia tips. In the mechanotransduction deficient IHC mutants *Tmie*^*KO/KO*^ and *Tmc1*^*KO/KO*^, BAIAP2L2 expression is very sparse and not enriched at any stereocilia location. In *Loxhd1*^*Delta/Delta*^, a strong BAIAP2L2 signal is detected in row 2 stereocilia but with a different distribution than control *Loxhd1*^*Delta/+*^ littermates. Boxes correspond to magnified panels. Scale bars = 2 μm. **b,** Stereocilia classification based on the BAIAP2L2 fluorescence signal distribution pattern: only at the tip, at the tip and along the shaft, at the shaft, or absent from stereocilia. Data are represented as mean ± SEM. Per group: n_mice_ ≥ 2; n_cells_ ≥ 8; Mann–Whitney test, two-tailed (see Extended Data Table 1). **c,** SUB-immunogold-SEM anti-BAIAP2L2 shows the enrichment of gold beads at the tips of rows 2 and 3 P21 IHC stereocilia in *Loxhd1*^+/+^, while gold beads are more distant from the tip in *Loxhd1*^*Delta/Delta*^. Arrowheads indicate the gold beads closest to the tips. Scale bars = 500 nm. **d-e**, Distance between BAIAP2L2-gold beads to tips of rows 2 (d) and 3 (e) stereocilia measured from SUB-immunogold-SEM micrographs in *Loxhd1*^+/+^ and *Loxhd1*^*Delta/Delta*^ P21. IHCs corresponded to ~70% apical, ~12% medial, and ~18% basal. Per group: P21 row 2 (d) n_mice_ ≥ 2; n_cells_ ≥ 34; n_stereocilia_ ≥ 168; n_gold_ ≥ 438; row 3 (e) n_mice_ ≥ 2; n_cells_ ≥ 34; n_stereocilia_ = 155 for *Loxhd1+/+* and 4 for *Loxhd1Delta/Delta*; n_gold_ = 303 for *Loxhd1+/+* and 4 for *Loxhd1*^*Delta/Delta*^ (see Extended Data Table 1). **f-g**, Stereocilia classification based on the BAIAP2L2-gold distribution pattern: present in the stereocilia, with at least one gold bead in the first 100 nm, with at least one gold bead below 100 nm, and without any gold beads. Row 2 (f): per group: n_mice_ ≥ 2; n_cells_ ≥ 33; n_stereocilia_ ≥ 289; row 3 (g): per group: n_mice_ ≥ 2; n_cells_ = 32; n_stereocilia_ = 280 for *Loxhd1*^+/+^ and 70 for *Loxhd1*^*Delta/Delta*^ (see Extended Data Table 1).

In P21 *Loxhd1*^*Delta/Delta*^ IHCs, TMC1 molecules were not confined to stereocilia tips and could instead be found mislocalized along stereocilia shafts. To confirm this striking result, we investigated the localization of BAIAP2L2 protein in stereocilia. BAIAP2L2 is a member of the membrane binding I-BAR protein family and localizes at and slightly below transducing stereocilia tips^50^. The maintenance of BAIAP2L2 localization at the stereocilia tips requires the presence of MET channels and is abolished by channel blockage^50^. We therefore predicted that BAIAP2L2 molecules should follow TMC1 mislocalization in the absence of LOXHD1. By previous reports^50–52^, our P21 IHC immunofluorescence staining in the control animals with a validated BAIAP2L2 antibody showed strong punctiform staining at the tips of row 2 and likely row 3 stereocilia, while BAIAP2L2 staining was instead sparse and not spatially restricted in IHCs from the *Tmc1*^*KO/KO*^ and *Tmie*^*KO/KO*^ mice (Fig. 4a). However, the BAIAP2L2 staining pattern in *Loxhd1*^*Delta/Delta*^ IHCs differed from these MET channel complex mutants. First, some strong staining was still detected in row 2 stereocilia, but the proportion of stereocilia with a stained tip was drastically reduced (Fig. 4a–b). Second, BAIAP2L2 staining could be found ectopically along stereocilia shafts, sometimes wrapped around their widths (Fig. 4a–b). To inspect nanoscale BAIAP2L2 stereocilia distribution, we performed SUB-immunogold-SEM (Fig. 4c). In P21 control (*Loxhd1*^*+/*+^) IHCs, the BAIAP2L2-gold beads were enriched within the first 100 nm from the tip (57% for row 2 and 98% for row 3) and found below the first 100 nm to a lesser degree in row 2 (Fig. 4c–e). Within the first 100 nm, BAIAP2L2 showed a bell-shaped distribution centered at 35 nm from row 2 tips and at 30 nm for row 3 tips (Fig. 4c–e). BAIAP2L2’s distribution in transducing stereocilia matched TMC1’s enrichment within the first 100 nm from stereocilia tips, with the difference being that TMC1 was asymmetrically distributed in this zone, found highest at the tip and fading rapidly after the first 50 nm (Fig. 3b). In P21 IHCs without LOXHD1 (*Loxhd1*^*Delta/Delta*^), BAIAP2L2 was no longer enriched within the first 100 nm of row 2 tips (21%), but instead distributed broadly within the first micron below the tip and almost absent from row 3 (Fig. 4d–e). The proportion of row 2 stereocilia with the BAIAP2L2 gold beads within the first 100 nm was drastically reduced (−67%), and the gold beads were largely redistributed below this region (Fig. 4f). Meanwhile, the proportion of row 3 stereocilia with the BAIAP2L2 gold beads was reduced by 90% (Fig. 4g).

Overall, we conclude that in the absence of LOXHD1, BAIAP2L2 is maintained at high levels in stereocilia but relocated away from the stereocilia tips, paralleling and confirming TMC1’s mislocalization.

### LOXHD1 interacts *in vitro* with TMC1 but not with TMC2

**Figure 5:**
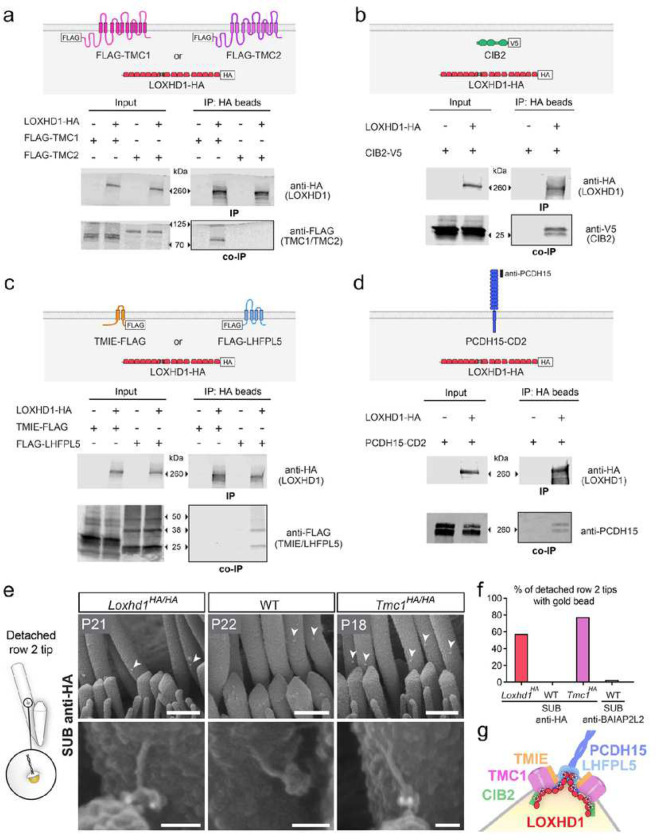
LOXHD1 interacts *in vitro* the channel complex and the tip link proteins and is connected to them *in vivo.* **a-d**, Co-immunoprecipitation experiments from HEK293t cells overexpressing LOXHD1-HA with tagged channel complex or tip link proteins. FLAG-TMC1 is co-immunoprecipitated with LOXHD1-HA using anti-HA magnetic beads, but FLAG-TMC2 is not (a); CIB2-V5 (b), FLAG-LHFPL5 (c) and PCDH15 (d) were co-immunoprecipitated with LOXHD1-HA, while TMIE-FLAG was not (b). Each experiment was replicated at least thrice; (a) was replicated five times. Predicted protein sizes based on primary sequence: LOXHD1-HA: 237 kDa; TMC1-FLAG: 88 kDa; TMC2-FLAG: 104 kDa; CIB2-V5: 23 kDa; TMIE-FLAG: 18 kDa; FLAG-LHFPL5: 25 kDa; PCDH15: 216 kDa. IP: Immunoprecipitation. **e**, In SUB experiments on P21 IHCs, some row 2 tips are detached from the rest of the stereocilia but still connected to the tip links (arrowheads and high magnification). Anti-HA gold-beads were specifically found at detached row 2 tips for LOXHD1-HA and TMC1-HA but not in the WT sample. The lower panels are high magnification of detached row 2 tips. Scale bars: 500 nm for the low mag pictures, 50 nm for the high mag ones. **f**, Quantification of the percentage of P21 IHC detached row 2 tips containing gold. Loxhd1HA/HA anti HA: n_mice_ = 2, n_cells_ = 17, n_detached tip with gold / total_ = 31/54; WT anti-HA: n_mice_ = 1, n_cells_ = 5, _ndetached tip with gold / total_ = 0/11; Tmc1HA/HA anti HA: n_mice_ = 2, n_cells_ = 10, n_detached tip with gold / total_ = 34/44; WT anti-BAIAP2L2: n_mice_ = 2, n_cells_ = 14, n_detached tip with gold / total_ = 1/42. **g**, Cartoon summarizing interactions between LOXHD1 and the MET channel complex and tip link. Note that LOXHD1 does not interact with TMIE.

We next wanted to understand the molecular mechanism by which LOXHD1 could facilitate the proper localization of TMC1 at the stereocilia tip. The fact that LOXHD1 selectively affects TMC1 localization but not TMC2, or LHFPL5 and only partially affects TMIE (Fig. 2 and Extended Data Fig.4) suggests that mislocalization is unlikely to be due to a general effect. Hence, we tested whether LOXHD1 could interact physically with TMC1 but not with TMC2 despite their strong homology (58% aa identity and 74% aa similarity). To test protein–protein interactions, we employed biochemistry. We single or co-transfected HEK293T cells with tagged full-length LOXHD1 and tagged candidate interactor constructs. After 48 hours of culture, we used the cell extracts for co-immunoprecipitation (co-IP) assays, which were subsequently run on SDS-PAGE gels and analyzed with tag-specific antibodies on western blots. When FLAG-TMC1 or FLAG-TMC2 was co-expressed with LOXHD1-HA and protein extracts pulled down by the HA-tag on LOXHD1, TMC1 co-immunoprecipitated, whereas TMC2 did not (Fig. 5a). The TMC1 interaction with LOXHD1 was further confirmed when FLAG-TMC1 was immunoprecipitated (Extended Data Fig. 7a). These experiments demonstrate that LOXHD1 interacts selectively *in vitro* with TMC1 but not with TMC2.

### LOXHD1 interacts *in vitro* with CIB2 but not with TMIE

Next, we similarly tested if LOXHD1 could interact with other known MET channel subunits CIB2 and TMIE, both of which are required for the presence and function of TMC1 in the HB ^5,7–11,31^. When LOXHD1-HA and CIB2-V5 were co-expressed and protein extracts pulled down by LOXHD1-HA, CIB2 was co-immunoprecipitated (Fig. 5b). Reversely, LOXHD1 was co-immunoprecipitated with CIB2 in the CIB2-V5 immunoprecipitation experiment, providing additional evidence for the interaction (Extended Data Fig. 7b). Contrarily, when FLAG-TMIE was co-expressed with LOXHD1-HA, TMIE did not co-immunoprecipitate along with LOXHD1 (Fig. 5c). The data demonstrate that LOXHD1 interacts *in vitro* with CIB2 but not with TMIE.

### LOXHD1 interacts *in vitro* with LHFPL5 and the lower tip-link protein PCDH15

We found that LOXHD1 can interact with the MET channel complex proteins TMC1 and CIB2, but not with TMC2 or TMIE. We next wanted to test whether LOXHD1 could also interact with LHFPL5, which is critical for force transmission from the tip link to the MET channel and can itself bind to TMC1 and TMIE and the tip link protein PCDH15 ^10,12,13^. When LHFPL5-FLAG was co-expressed with LOXHD1-HA in HEK293T cells and protein extracts were pulled down by LOXHD1-HA, LHFPL5 was co-immunoprecipitated with LOXHD1 (Fig. 5c). Conversely, LOXHD1 co-immunoprecipitated along with FLAG-LHFPL5, confirming their interaction (Extended Data Fig. 7a). Finally, we wondered whether LOXHD1 could interact with the lower tip-link protein PCDH15. We focused on PCDH15-CD2, the only PCDH15 isoform indispensable for hearing in mice^53,54^. When PCDH15-CD2 was co-expressed with LOXHD1-HA and cell extracts pulled down by LOXHD1-HA, PCDH15 was co-immunoprecipitated (Fig. 5d). The interaction was also confirmed upon PCDH15 immunoprecipitation (Extended Data Fig. 7d).

In conclusion, LOXHD1 interacts selectively with three of the five known MET channel complex proteins TMC1, CIB2, and LHFPL5 and with the tip link itself.

### LOXHD1 is connected to the tip link and the MET channel complexes *in vivo*

To determine the precise localization of LOXHD1 at the tips of transducing stereocilia, we took advantage of SUB-immunogold-SEM and performed it on P21 *Loxhd1*^*HA/HA*^ hair cells. The LOXHD1-gold beads localized to the tips of row 2 and 3 stereocilia of IHCs and OHCs and more rarely along the length of row 1 stereocilia (Extended Data Fig. 8a). Interestingly, in the preparations done at this age, tip links joining rows 1 and 2 of IHCs were frequently detached at their lower insertion side but remained attached at their upper side to the tallest stereocilia (Fig. 5e). Extra material from the tips of row 2 was consistently attached to the tip links, indicating that the breaking points were located deeper than the site of lower tip-link membrane insertion. The LOXHD1-gold beads were frequently found attached to this externalized lower tip-link insertion material (57% of detached row 2 tips) (Fig. 5e–f). The LOXHD1 gold beads appeared positioned below the tip link branches (Fig. 5e). Conversely, when the experiment was performed on non-HA animals, no gold was detected on the detached lower tip-link insertion material (Fig. 5e–f). When anti-HA SUB-immunogold-SEM was done on hair cells from *Tmc1*^*HA/HA*^ mice, the gold beads were also frequently attached (77% of detached row 2 tips), demonstrating the existence of a physical connection between TMC1 and the tip link. By contrast, when anti-BAIAP2L2 SUB was performed on the P21 WT animals, the gold beads were rarely found (2%) at the detached row 2 tips (Fig. 5f and Extended Data Fig. 8b). Thus, we conclude that as for TMC1, some LOXHD1 proteins are physically connected to the lower tip-link branches and the MET channel complexes *in vivo*.

In summary, LOXHD1 is an indispensable molecular connector between the tip link and the MET channel complex, required to confine and maintain TMC1 molecules at this nanometric site of the stereocilia membrane (Fig. 5g). Without LOXHD1, TMC1 molecules become progressively mislocalized, preventing their directional mechanical activation by sound-induced forces. This phenotype represents a novel molecular mechanism causing hearing loss in vertebrates.

## Discussion

Here, we demonstrate the existence of an unanticipated critical element of the vertebrate auditory MET machinery: an internal connector that couples the tip link to the ion channels. Without LOXHD1, the MET channel subunit TMC1 is not maintained within proximity to the tip link. Force is not efficiently transferred from the tip link to the MET channel; therefore, the auditory MET channels including TMC1 cannot be activated by sound, leading to hearing loss.

### TMC1 MET is selectively affected by the absence of LOXHD1

We found that two phenotypes occur in cochlear hair cells without LOXHD1. First, the quantities of TMC1 molecules are reduced in stereocilia, suggesting that LOXHD1 may regulate TMC1 expression levels, stereocilia targeting, or maintenance. This phenotype strongly affects row 3 stereocilia of homozygous *Loxhd1*^*Delta/Delta*^ IHCs and OHCs and precedes morphological defects in stereocilia (width and maintenance). Importantly, while TMC1 expression is not affected in heterozygous *Loxhd1*^*Delta/+*^ IHC at P21, it is reduced in OHC already at P7, likely explaining the partial hearing loss of the heterozygous animals. Second, in the absence of LOXHD1, the remaining TMC1 molecules progressively lose their stereocilia tip enrichment, creating a mismatch between potentially mechanically activatable TMC1 channels and the force-receiving sites near the tip links. At P7, when TMC2 is present and contributes to half of the current^14^, the absence of LOXHD1 would affect only TMC1 (32% row 2 tips with TMC1 remaining) (Fig. 3h). Therefore, the predicted max MET current would be 354 pA in agreement with the measured 357 ± 54 pA (Fig. 1h–i). At P11, IHC MET currents are reduced by 84% in the absence of LOXHD1 from 412 ± 60 pA to 66 ± 46 pA (Fig. 1j–k). If we consider that rows 2 and 3 contribute equally to these currents^17^, then we would predict that in the absence of LOXHD1, 42% of row 2 tips and 15% of row 3 tips with TMC1 would generate a total of 117 pA of MET maximum currents (Fig. 4h and 4i). This prediction is about one SD of the mean of the measured current, largely explaining the MET current phenotype. At P21, based on TMC1 SUB localization, only 2% of the MET current would be left, preventing hair cell depolarization by sound and leading to hearing loss. Overall, in the absence of LOXHD1, the MET currents of channels containing TMC1 are affected, while those containing TMC2 seem not to be. These results are supported by the fact that the proportion of row 2 tips with TMC2 is unaffected in *Loxhd1*^*Delta/Delta*^ (Fig. 2c and 2d) and that TMC2 cannot bind LOXHD1 *in vitro* (Fig. 5a). This later result is striking as both proteins are closely related, and we will determine in the following studies which domains create this distinction.

Overall, LOXHD1 is a novel element of the auditory MET complex, which is exclusive to a given TMC paralogue, implying that further differences could exist between the TMC1 and TMC2 channels, such as distinct interactions with the cytoskeleton, tip link, or membrane, and that these distinct channels could potentially have different channel gating mechanisms and activity regulations as well.

### The mature auditory MET channel complex requires a 5th subunit to maintain coupling to the tip link

Previous *in vitro* and electrophysiological studies have reported the existence of multiple molecular interactions between the pore channel subunit TMC1 and the lower part of the tip link, PCDH15: TMC1 can interact directly with PCDH15^33^ or indirectly via TMIE and LHFPL5, which can also interact between themselves^10–12,31,32^. However, we found that the combination of all these interactions is insufficient to maintain TMC1 connected to the tip link in the absence of LOXHD1. Furthermore, by determining how a given subunit is affected by this TMC1-PCDH15 decoupling, some insights about the strengths of their mutual *in vivo* interactions can be deduced from our experiments with *Loxhd1*^*Delta*^ mutant hair cells: Because LHFPL5 is maintained at the tip of stereocilia while TMC1 is mislocalized at P11, the interaction between LHFPL5-PCDH15 appears stronger than the LHFPL5-TMC1 interaction. Therefore, LHFPL5 is integral to the lower tip link, as suggested by previous *in vitro* data^13,35^. Contrarily, as TMIE is partially removed from stereocilia tips while TMC1 gets mislocalized at P11 in *Loxhd1*^*Delta/Delta*^, a TMIE pool must bind equally or more strongly to TMC1 than to PCDH15 and LHFPL5.

Overall, by binding to three MET channel complex subunits and the tip link, we propose that LOXHD1 provides cohesiveness and stability to the channel complex–tip link coupling. Therefore, while the multi-subunit nature of the MET channel complex is important for providing different modes of activity regulations, like TMIE for PIP2^11^ and CIB2^9^ for its potential interaction with cytoskeletal proteins, its multi-subunit nature is also a weakness. The mature MET machinery requires a dedicated mechanism to maintain the cohesion and integrity of the channel complex and its coupling to the tip link to remain functional. As the auditory MET channels can be disengaged from the mechanical stimulation *externally* by the tip link rupture, we found that they can also be disengaged *internally* by a physical decoupling from the tip link. Besides these discoveries, our work demonstrates that auditory channels can be disconnected from the tip links while being maintained in the stereocilia membrane at least until P21. Their presence likely explains the maintenance of the stereocilia themselves. Our findings also open the exciting possibility that the mislocalized channels could be reconnected to the tip link even at a late stage, potentially allowing for therapeutic intervention to restore MET function and hearing of the *Loxhd1*-deficient mice and, ultimately, human DFNB77 patients. However, some roadblocks will have to be overcome to test this hypothesis as the *Loxhd1* coding sequence is large and requires a dual AAV approach, as functional restoration could involve different splicing isoforms, and as the overexpression of *Loxhd1* can have deleterious effects on cells, such as by driving Ewing’s sarcoma cells’ malignancy^55^.

### Nanoscale mapping of the auditory machinery in stereocilia

LOXHD1 and the other subunits of the MET channel complex are present at low levels in stereocilia. To track these endogenous proteins in stereocilia at nanoscale resolution, we used our recently developed SUB-immunogold-SEM method (Miller et al, *submitted*). This method allows the imaging and mapping of gold bead conjugated antibodies to detect internal epitopes along cell surfaces. Most importantly, this technique permits fast sampling of large sample numbers, providing high accuracy and sensitivity to our quantifications. For instance, TMC1 distribution analysis in WT IHC HB (P7, P11, and P21) integrated data from 56 cells, 459 stereocilia, and 646 gold beads. This method opens new possibilities for HB investigations from embryonic to adult stages, such as the identification of minute but functionally relevant alterations in protein localization, or in determining protein localization in small row 3 stereocilia.

In this study, we precisely mapped the TMC1 subunit of the auditory MET channel complex. While TMC1 has been previously localized at the tip of stereocilia by fluorescence and even by immunogold-TEM microscopy, the distribution of TMC1 molecules relative to the stereocilia tip could not be measured ^29,38,56^. Here, we showed that TMC1 molecules are concentrated at the extreme tip of stereocilia, contained in the first 100 nm, and concentrated further within the first 50 nm for row 2 and 3 stereocilia of IHCs and OHCs. These measurements were from fixed, dehydrated, and metal-coated samples. To convert them to living measurements, we can take advantage of the scaling factors determined in our previous study ^57^, giving an estimated TMC1 concentration within the first 83 nm from tips of living transducing stereocilia. Given their close proximity to the tip link, we hypothesize that at least some TMC1 channels may be mechanically activated by membrane stretching ^58^. Furthermore, we provide the first *in situ* evidence that TMC1 molecules are physically connected to the internal part of the lower tip link insertion point (Fig. 5e–f). LOXHD1 is also attached to this complex and appears to be below the tip link branches. We provide strong evidence here that LOXHD1 is an integral element of the auditory MET machinery, connecting the tip link and TMC1. LOXHD1 is in a prime position to contribute to the force transmission and modulation of auditory channel activity. These hypotheses, as well as the systematic mapping of the other MET complex subunits in stereocilia at the nanoscale with SUB-immunogold-SEM, will be explored in further studies.

### Limitations of the study

Given that the best tool available to track CIB2 is an antibody that has a high background level, we could not determine with accuracy if CIB2 was affected by LOXHD1’s absence. A tagged KI animal would be ideal to answer this question, but finding a location in this small protein for tag insertion and maintaining its function is challenging.

## METHODS

### Experimental models

#### Mouse strains

The Administrative Panel on Laboratory Animal Care (APLAC) at Stanford University (APLAC protocols #28278 and #30305) approved all animal procedures. Mice of both sexes were used in all experiments. The mice were housed in standard Innovive cages with bedding (San Diego, CA) under 12 hours light/dark cycles with permanent access to food and water, with room temperature (RT) maintained at around 22°C. The mice were also inspected weekly for any signs of discomfort, and the sentinel mice were regularly tested for infection on each cage rack. *Tmc1*^*HA*^, *Tmc2*^*Myc*^, and *Tmie*^*HA*^ were obtained from Dr. Cunningham and Dr. Müller (J. Hopkins University) ^11^, *Tmie*^*KO* 59^ (B6.B(CBA)-*Tmie*^*sr*^/J, JAX 000543) from JAX, and *Tmc1*^*KO* 1^ (B6.129-*Tmc1*^*tm1.1Ajg*^/J, JAX 019146, backcrossed on C57Bl6/J) from Dr. Beurg and Dr. Fettiplace (Wisconsin University). The mice were genotyped as previously described, and the C57BL6/J WT mice were purchased from Charles River Laboratories.

#### *Loxhd1*^*Delta*^ and *Loxhd1*^*HA*^ *Mouse Generation*

The *Loxhd1*^*Delta*^ and *Loxhd1*^*HA*^ alleles were generated by CRISPR/Cas9 gene editing. sgRNA target sites were selected using the CRISPRscan (crisprscan.org) or CRISPick (Broad Institute) websites. For the *Loxhd1*^*Delta*^ allele, the original design was meant to generate a floxed allele with a direct recombination reporter (mScarlet) like the previous design^60^. An sgRNA target site located between Exon 1 (gG18NGG-64) and Exon 2 was selected to insert *LoxP/FRT* elements, and a second sgRNA target site after the last exon (Exon 41) was selected to insert *LoxP/SA/mScarlet/FRT* elements. sgRNAs were synthesized from DNA templates by *in vitro* transcription with the HiScribe T7 High Yield RNA Synthesis Kit (NEB, E2040S). Each sgRNA DNA template was produced by PCR using a specific sense primer that contains a T7 promoter and an sgRNA target sequence and an antisense primer amplification on the pX461 vector (https://www.addgene.org/48140/) that contains a common sgRNA sequence. Single-stranded oligodeoxynucleotides (ssODNs) for inserting desired elements were synthesized by using the Guide-it Long ssDNA Production System (Takara, 632644). A mix of the Cas9 nuclease protein (15 ng/μl, NEB, M0386S), 2 sgRNAs (15 ng/μl for each), ssODN for *LoxP/FRT* (2 ng/μl), and ssODN for *LoxP/SA/mScarlet/FRT* (8 ng/μl) diluted in nuclease-free injection buffer (10-mM Tris-Cl, 0.1-mM EDTA, pH 7.4) was used for pronuclear injection into one-cell C57BL/6J embryos. The steps of *in vitro* fertilization, pronuclear injection, and embryo transplantation to the pseudopregnant mice were performed by the Transgenic, Knockout, and Tumor Model Center (TKTC) of Stanford University. Mouse pups from the injected embryos were tail clipped before weaning, and genomic DNA was extracted using the DNeasy Blood & Tissue Kit (Qiagen, 69506). To determine the result of gene editing on each mouse, the genomic DNA was screened by PCR and sequenced. A mouse containing a deletion of a 161483 bp *Loxhd1* gene fragment was identified. The Sanger sequencing results affirmed that the deletion started at the first sgRNA target site (between Exons 1and 2) and ended at the second sgRNA target site (after Exon 41). A total of 859 bps from the second ssODN sequence containing part of the mScarlet CDS up to the FRT site was inserted between the two sgRNA target sites. This allele, named *Loxhd1*^*Delta*^, corresponds to the deletion of a 161483 bp in the *Loxhd1* gene (from chr18:77282757 to chr18:77444193, GRCm38/mm10), removing the protein-coding Exons 2 to 41. Germline transmission was confirmed by breeding the founder mice with the C57BL/6J mice and backcrossing for five generations before the phenotyping analysis.

For the *Loxhd1*^*HA*^ allele, a 30 bp DNA fragment coding a glycine linker and an HA-tag (YPYDVPDYA) was inserted in Exon 1of *Loxhd1* between the codons for the 16th amino acid, lysine, and 17th amino acid, glycine, as they show less evolutionary conservation than the rest of the protein. The chemically modified sgRNA (Gg18NGG-39, CRISPRScan) was ordered from Synthego (Redwood city, CA). A 127 bp asymmetric ssODN ^61^ containing the 30 bp tag insertion, flanked by a 36 bp homology arm on the PAM-distal side and a 61 bp homology arm on the PAM-proximal side complementary to the nontarget strand, was synthesized by IDT (Coralville, IA). A mix of Engen Cas9 NLS (10 ng/μl, NEB, M0646T), sgRNA (10 ng/μl, Synthego), and ssODN (20 ng/μl, IDT) diluted in nuclease-free injection buffer was injected into the pronucleus of one-cell C57BL/6J embryos. The steps of *in vitro* fertilization, pronuclear injection, and embryo transplantation to the pseudopregnant mice were performed by the TKTC of Stanford University. The genomic DNA of founder animals was screened by PCR and sequencing. Germline transmission was verified by breeding the founder mice with the C57BL/6J mice, and the mice were backcrossed for at least two generations before phenotyping analysis. Genotyping was performed routinely by PCR screening and an established qPCR protocol with automatized Transnetyx genotyping service (Cordova, TN). The genotypes and ages of mice used in specific experiments are indicated in the Fig.s and the main text.

#### Cell Lines

The HEK293T cell line from ATCC (#CRL-3216) was used for heterologous expression. The cells were cultured in DMEM + GlutaMax (Thermo Fisher, 10566016) with 10% fetal bovine serum and 1% *Penicillin*/*Streptomycin* added. The cells were maintained at 37°C with 5% CO_2_ and were passaged at 80% confluency.

## METHOD DETAILS

### Auditory measurements

The P21 mice were anesthetized with an intraperitoneal (IP) injection of 100-mg/kg ketamine (VetaKet CIII, AKORN, NDC 59399–114-10) and 12.5-mg/kg xylazine (AnaSed injection, AKRON, NDC 59399–110-20), and the P30 mice were anesthetized with 100-mg/kg ketamine / 12.5-mg/kg xylazine / 2.5-mg/kg acepromazine (VetOne, NDC 13985–587-50). The anesthetized mice were placed on a 37°C heating pad during and after recording until they had fully recovered.

The closed field tone ABRs were tested as previously described^44^. The RZ6 Multi I/O Processor (RZ6-A-P1, TDT, Tucker-Davis Technologies, Alachua, FL), the RA4PA standard Medusa Preamplifier (TDT), a RA4LI low-impedance headstage (TDT), a needle electrode kit (ELE-N, TDT), and high-frequency speakers (MF1, TDT) were set up on the basis of the customer guide of TDT. The ABR testing system was controlled by BioSigRZ (TDT). Before each experiment, the MF1 speaker was calibrated with a 1/4” free field microphone (PCP Piezotronics, Depew, NY). Moreover, the recording needle electrode was inserted under the skin on the vertex of the head, the reference electrode was inserted below the ipsilateral ear with a stimulating speaker, and the ground electrode was inserted below the contralateral ear. Five-millisecond tone pip stimuli were examined across a range of frequencies, including 4, 5.7, 8, 11.3, 16, 22.6, 32, and 45.3 kHz, at sound pressure levels (SPL) ranging from 0 to 80 dB in increments of 10 dB. The signals were filtered with 3-kHz low pass and 300-Hz high pass filters, along with a 60-Hz notch filter. The presentation rate was 21/sec, and 512 trails of 10-ms ABR were averaged. The ABR thresholds were defined as the lowest sound intensity at which the wave corresponding to the first evoked auditory potential could be reliably discerned. (NR: non-responsive). Distortion products of otoacoustic emissions (DPOAEs) were tested as previously described^44^. Two high-frequency speakers (MF1, TDT) connected to the RZ6 Multi I/O Processor were used to deliver two simultaneous continuous pure tones (*F1* and *F2*), and a probe-tip microphone (type 4182, Bruel & Kjaer, Nærum, Denmark) connected to a condition amplifier (Nexus, Bruel & Kjaer) was used to detect DPOAEs from the external auditory canal. Before each experiment, MF1 speakers were calibrated with 1/4’’ free field microphone (PCP Piezotronics, Depew, NY). Two 1-s sine wave stimulus tones, each with different frequencies (F1 = 0.909 of center frequency and F2 = 1.09 of center frequency), were presented at equal intensity levels. These stimuli ranged from 0 to 80 dB SPL in 10 dB increments and were centered around frequencies of 4, 5.7, 8, 11.3, 16, 22.6, 32, and 45.3 kHz. The amplitude of the cubic distortion product was detected at *2F1-F2*. DPOAE thresholds corresponded to the sound intensity at which the amplitude of the cubic distortion product 2f1-f2 was 2 SDs above the noise floor.

### Scanning electron microscopy (SEM)

SEM sample preparation and imaging were conducted as described in detail in ^62^. In brief, the inner ears were isolated in dissection buffer (DB) (1 X HBSS with Ca^2+^ and Mg^2+^, with osmolarity adjusted to 310 mOsm with D-glucose) and fixed in 4% PFA diluted from 32% stock in this buffer for 30 min at RT. The inner ears were then dissected to remove bone structures, the stria vascularis, and Reissner’s and tectorial membranes. The samples were refixed in 2.5% glutaraldehyde and 4% PFA in DB overnight at 4°C, washed, dehydrated in ethanol (30%, 50%, 75%, 95%, 100%, and 100%, 5 min incubations), and processed to the critical drying point using Autosamdri-815A (Tousimis). The cochleae were mounted on studs using silver paint and coated with 4-nm palladium (sputter coater EMS150TS, Electron Microscopy Sciences). The samples were imaged with a 5-kV accelerated voltage and a 13-pA beam current using a secondary electron detector on an FEI Magellan 400 XHR Field Emission Scanning Electron Microscope at the Stanford Nano Shared Facilities. The microscope is periodically calibrated for measurements using a SIRA-type calibration specimen for ultra-high-resolution modes with 2% error between 50- and 350-k magnification at our imaging settings.

### Quantification of Hair Bundle Morphology

To compare HB morphology between WT control and the *Loxhd1*^*Delta/Delta*^ mice, we quantified different stereocilia parameters from SEM micrographs using ImageJ2 software^63^. For IHCs, we quantified the number of stereocilia for rows 1–3, as well as their widths, which was measured at their widest position.

For OHCs, we approximated the heights of rows 2 and 3 stereocilia from OHCs that were imaged close to the orthogonal plane with respect to the HB face from the 200–300th most apical OHCs (counted from low magnification SEM pictures). On the basis of 726 IHCs found along the entire adult mouse cochlea^1^, we imaged the 27%–41% most apical OHCs across development, which are expected to contribute to the 10.7- to 14-kHz characteristic–frequency range in adults ^64^. As an approximation of the absolute stereocilia height, we measured the height of row 3 and added the rows 2 to 3 step to obtained row 2 height. The coefficient of variation of the stereocilia height was determined by dividing the standard deviation by the mean and was expressed as a percentage.

To evaluate the morphological changes of the IHC row 2 stereocilia tips between the *Loxhd1*^*Delta/Delta*^ and WT mice, we traced the contours of the tip region from SEM images with Illustrator (Adobe). The same age and genotype stereocilia tip contours were aligned and stacked according to the symmetrical midlines and vertices while maintaining the scaling value.

### Electrophysiology

The IHCs were recorded using a whole-cell voltage clamp as previously described ^44^. All the experiments were performed at RT (19°C–22°C), on the apical cochlear turn, corresponding to the 6- to 10-kHz frequency range in the adult mouse.

The tissue was dissected and perfused with an extracellular solution containing the following: 145-mM NaCl, 2-mM KCl, 2-mM CaCl_2_, 1-mM MgCl_2_, 10-mM HEPES, 6-mM glucose, 2-mM pyruvate, 2-mM ascorbic acid, and 2-mM creatine monohydrate. The pH of the external solution was adjusted to 7.4, and the osmolarity ranged from 309 to 310 mOsm. We used borosilicate patch pipettes with a resistance of 2.5–3.5 MΩ, loaded with an internal patch solution containing the following: 116-mm CsCl, 3.5-mM MgCl_2_, 3.5-mM ATP, 5-mM creatine phosphate, 0.1-mM tetracesium BAPTA, 10-mM HEPES, and 20-mM ascorbic acid (pH 7.2, 290 mOsm).

The MET currents were low-pass filtered at 100 kHz, measured with an Axopatch 200B patch-clamp amplifier, digitized with a daq3000 instrument (IOtech) at 500 kHz, and recorded using jClamp. For whole cell recording, the cells were held at −80 mV. The uncompensated series resistance was <10 MΩ, and cells with >100 pA of leak current were discarded. For the fluid jet stimulation of HBs, we used a piezoelectric disk bender delivering the fluid stimulation via a pipette with a diameter of 10–14 μm. We also used 1 kHz with an 8-pole Bessel filter (frequency devices) to filter the voltage stimulus (600–1600 mV).

### Dissection and fixation of the Organ of Corti

The temporal bones were removed from the skull and put in a dish with ice cold dissection buffer (1 × HBSS with Ca^2+^ and Mg^2+^, with osmolarity adjusted to 310 mOsm with D-glucose) as described in detail in^65^. The inner ears were then dissected out and transferred to a dish with a fixative (4% PFA in dissection buffer), a hole was poked on the bony cochlear shell at the apex, and the fixative was perfused slowly through round and oval windows. The perfused inner ears were incubated in the fixative for 40 min (20 min for the TMIE-HA samples) at RT.

### Whole-mount immunofluorescence staining and imaging

The whole-mount immunofluorescence staining and imaging of the mouse cochlear HBs in this study followed our previously published detailed protocol^65^. Briefly, the fixed inner ear samples were transferred to new dishes with 1 × PBS, and the bony cochlear shell, stria vascularis, Reissner’s membrane, tectorial membrane, and the modiolus were sequentially removed. The finely dissected organs of Corti were transferred to a glass well plate with PBS containing 0.05% Triton X-100 and permeabilized for 40 min at RT. After permeabilization, the samples were blocked in PBS with 0.05% Tween 20 (PBST) containing 4% bovine serum albumin Fraction V (BSA) overnight or at least 6 hours at 4°C. The tissues were then incubated with primary antibodies in PBST with 1% BSA (incubation buffer) overnight at 4°C. The samples were then washed 4 times, 5–10 min per wash, in the incubation buffer at RT. Subsequently, the tissues were incubated with fluorescent dye-conjugated secondary antibodies (see below) in the incubation buffer at RT for 1–2 hours. After one wash with the incubation buffer, the samples were incubated with fluorescent dye-conjugated phalloidin (see below) in incubation buffer at RT for 25 min. The samples were then washed thrice, 5–10 min per wash, with incubation buffer. For the TMIE-HA samples, the detergent of permeabilization buffer, blocking buffer, and incubation buffer was changed to 0.05% saponin. The glass well plate was on a horizontal shaker with a 60-rpm speed during permeabilization, incubation, and washing steps. For TMC1-HA, TMC2-MYC, TMIE-HA, and LOXHD1-HA, each experiment contained at least one parallel stained cochlea sample from a similar age mouse without any tag as a background control. After washing, each sample was mounted on a glass slide under a coverslip by using ProLong Gold Antifade Mountant (Thermo Fisher Scientific). Z-stacks were captured using the Airyscan Super-resolution mode of a Zeiss LSM880 microscope with Objective C Plan-Apochromat 63x/1.4 Oil DIC M27 lens and Zen black software (Zeiss). The image acquisition parameters were determined as the best X*Y and Z axis resolution possible for the shortest wavelength used channel.

### Immunofluorescence image processing and quantification

Zeiss Zen software (blue edition, version 2.3) was used to process the Airyscan CZI format images. The processed CZI format images were converted into an Imaris file (IMS) format by ImarisFileCoverter (Oxford Instruments version 9.8.0 or version 9.9.1), opened, and 3D reconstructed with Imaris (Oxford Instruments, version 9.8.0 or version 9.9.1 or version 10.0.0). To identify the IHC row 2 stereocilia tip signal of target proteins, the surface tool of Imaris was used to generate the 3D rendering of each channel by utilizing the automatic algorithm without smoothing the surfaces. The “background subtraction” option was selected for thresholding with the following parameters: (1) the “diameter of the largest sphere fitting into a 3D object” was set to 265 nm; (2) for the phalloidin channel, the threshold was set at 10% of its highest intensity; (3) for HA and MYC tagged proteins, the threshold was defined as the average intensity of the same fluorescent channel on row 2 stereocilia from the genetic negative control (no tag); (4) for LHFPL5, the threshold was defined as the average intensity of the row 1 region without puncta signals; (5) the minimal diameter defining an object or seed point was set to 200 nm; (6) the seed point was adjusted using the quality filter set at 18 for HA and MYC tagged proteins and at 300 for LHFPL5; and (7) the other software parameters were set to default values.

From the 3D model, a given puncta was scored as present at a row 2 tip when in contact with the first 400 nm of the stereocilia tip or when placed above the stereocilia tip with a maximum gap distance of 100 nm (see Movie and Fig. S3A). The percentage of row 2 tip with puncta was determined and compared between genotypes.

To quantify the TMC1-HA whole HB intensity of P7 OHCs, individual OHC HB volumes were manually segmented from the phalloidin signal on each Z stack. The sum intensity of TMC1-HA in each OHC HB volume was then determined. To combine data from different experiments, the average intensity of background control ($I_{\text{Background}}$) and the average intensity of apical *Tmc1*^*HA/HA*^; *Loxhd1*^+/+^ ($I_{\text{WT}}$) were used to normalize the intensity values of each hair bundle through the following equation:

$$I_{\text{Normalized}}=100*\left(I-I_{\text{Background}}\right)/I_{WT}\text{. }$$

The quantification of BAIAP2l2 immunofluorescence staining was based on the signal distribution pattern relative to the height of phalloidin-labeled row 2 stereocilia. The signal was categorized as either “above or at the row 2 tip” or “below this region,” with the latter being considered as a shaft signal; it was also noted when the signal was present at both locations or absent altogether. The percentage of each pattern was calculated per HB.

### SUB-immunogold-SEM labeling, processing, imaging, and quantification

The fixed inner ear samples were transferred to new dishes with dissection buffer to dissect the organs of Corti out. Thereafter, the samples were transferred to 2-ml tubes with TBST (150-mM NaCl, 10-mM Tris-HCl, 0.05% Tween-20, pH 7.5) containing 0.5% Triton X-100, permeabilized for 1 hour at RT. After permeabilization, the samples were washed with TBST once and then blocked in TBST containing 4% BSA for at least 6 hours or overnight at 4°C. The samples were transferred to 0.3-ml PELCO mini vials (TED PELLA, #21441) with primary antibodies in TBST with 1% BSA overnight at 4°C. Subsequently, the samples were transferred into 2-ml tubes, rinsed once, and washed thrice (15 min per wash) with 1% BSA TBST. The samples were then transferred to 0.3-ml PELCO mini vials with 10-nm gold conjugated goat anti-rabbit IgG (BBI: 1:200 in 1% BSA TBST) and incubated overnight at 4°C. After 2nd antibody incubation, the samples were rinsed once and washed thrice (15 min per wash) with 1% BSA TBST in 2-ml tubes. The samples were then rinsed twice with 0.1-M sodium cacodylate buffer (pH 7.2) and fixed with 10% glutaraldehyde, 4% PFA in 0.1-M sodium cacodylate buffer for at least 24 hours at 4°C, then washed with 0.1-M sodium cacodylate buffer, dehydrated in ethanol (30%, 50%, 75%, 95%, 100%, and 100%, 5-min incubations), and then processed to the critical drying point using Autosamdri-815A (Tousimis). The cochleae were mounted on studs using silver paint and coated with 2- to 3-nm of palladium (sputter coater EMS150TS, Electron Microscopy Sciences). The samples were imaged with a 5-kV accelerated voltage and a 100-pA beam current using a concentric backscattered electron detector on an FEI Magellan 400 XHR Field Emission SEM. The gold beads, characterized by their circular shape with an approximate diameter of 10 nm, were easily described as sources of backscattered electrons, and they were distinguishable from the signal originating from the stereocilia surface. The micrograph contrast was adjusted via Photoshop (Adobe) to display the gold better when needed. As stage tilting is impossible in backscattered electron imaging mode, HB orientation could not be adjusted for optimal imaging. The distance measurements of the gold to stereocilia tips were performed with ImageJ2 after scale calibration, placing the measuring ends at the center of the gold bead and at the pointiest location of the stereocilia tip. These distances were approximations of the absolute distances as we measured the shortest distance between the gold beads and the stereocilia tip on 2D pictures without accounting for the stereocilia volume and the perspective distortion of the images. For the gold bead distribution pattern, we determined the number of stereocilia in each HB with the following: at least one bead within the first 100 nm, at least one bead below 100 nm from the tip, at least one bead at any position, or without any bead.

### DNA constructs and plasmids

All plasmids were fully sequenced before use using the whole-plasmid sequencing service of Primordium Labs (Monrovia, CA). pCMV-hCIB2-V5 ^5^ was gifted by Dr. Zubair Ahmed at University of Maryland, and pN3-HA-mCIB2 ^8^ and pCMV-mPCDH15-CD2 ^37^ were from Dr. Ulrich Müller at John Hopkins University.

**pcDNA3-mLoxhd1(with CC)-MYC** was modified from pcDNA3-mLoxhd1(without CC)-MYC^44^. A coiled-coil (CC) encoding sequence containing fragment (Exons 18–21, with alternative CC Exons 19 and 20) was amplified from a P7 mouse cochlear cDNA library with PCR primer pair NG-230/NG-233. Similarly, an Exon 21–37 fragment was amplified with PCR primer pair NG-232/NG-229. Thereafter, the two fragments were hybridized and amplified by PCR with primer pair NG-230/NG-229, producing an Exon 18–37 fragment. Next, this Exon 18–37 fragment was integrated with pcDNA3-mLoxhd1(without CC)-MYC with Gibson Assembly (Gibson Assembly Master Mix (NEB, E5510S)). The Exon 18–37 fragment was amplified with PCR primer pair PW-40/PW-41 and pcDNA3-mLoxhd1(without CC)-MYC with PCR primer pair PW-42/PW-43.

NG-229, GCCAGGGTCCCACTGTCTCCATTTT

NG-230, GTCCACTACGAGATTGAGATTTGGACGG

NG-232, ACAGATACCTTCACCATCTATGCCATTGA

NG-233, GTGGTCTCGTTATTCACGTCGGTGATGT

PW-40, CCAGAAATTGGTCCACTACGAGATTGAG

PW-41, CTCCATCAATGACTGCACATATCTCACAC

PW-42, ATGTGCAGTCATTGATGGAGAGGAGATG

PW-43, CGTAGTGGACCAATTTCTGGATCTCCAC

**pcDNA3-mLoxhd1(with CC)-HA** was modified from pcDNA3-mLoxhd1(with CC)-MYC by replacing The Myc-tag with the HA-tag with HA containing PCR primer pair PW-85/PW-86 amplification and following *in vitro* assembly with the In-Fusion Snap Assembly Master Mix (Takara, 638948).

PW-85, TACCCATACGATGTTCCAGATTACGCTTGAGAATTCCACCACACTG

PW-86, TCTGGAACATCGTATGGGTAAACGGCCGCGACAGACGGGAAGAGCTC

**pN3-FLAG-mTmc1** was modified from pN3-HA-mTmc1 (a gift from Dr. Ulrich Müller at John Hopkins University) by replacing the HA-tag with the Flag-tag. FLAG-containing primer pair PW-139/PW-140 was used to amplify the mTmc1 CDS from pN3-HA-mTmc1. The amplified insert was digested with BglII/NotI, purified by electrophoresis, and gel extracted. The insert was ligated into BgIII/NotI digested pN3 backbone with T4 ligase (NEB, M0202S).

PW-139, ACTCAGATCTCGAGATGGATTATAAAGATGATGATGATAAAGCTCAAGCTTCGAATTCTGCAGTC

PW-140, TATGATCTAGAGTCGCGGCCGCT

**pN3-FLAG-mTmc2** was modified from pN3-MYC-mTmc2^37^ by replacing the the Myc-tag with the Flag-tag. FLAG-containing primer pair PW-141/PW-140 was used to amplify the CDS of mTmc2 from pN3-MYC-mTmc2. The amplicon was inserted into BglII/NotI digested pN3 backbone with the NEBuilder HiFi DNA Assembly Master Cloning Kit (NEB, E5520S).

PW-141, ACTCAGATCTCGAGATGGATTATAAAGATGATGATGATAAACAAGCTTCGAATTCTGCAGTCGA

PW-140, TATGATCTAGAGTCGCGGCCGCT

**pN3-mTmie-FLAG** was modified from pN3-mTmie-HA^10^ by replacing the HA-tag with the Flag-tag by FLAG-containing PCR primer pair PW-350/PW-351 amplification followed by *in vitro* assembly with the In-Fusion Snap Assembly Master Mix (Takara, 638948).

PW-350, GACTACAAAGACGATGACGACAAGTGAGCGGCCGCGACTCTAGATCATAAT

PW-351, CTTGTCGTCATCGTCTTTGTAGTCCATTTTCTCTCCTTTCTTCTTGGCCTC

**pcDNA3-FLAG-mLHFPL5** was modified from pcDNA3-HA-mTMHS (mLHFPL5)^13^ by replacing the HA-tag with the Flag-tag using FLAG sequence-containing PCR primer pair PW-348/PW-349 amplification and *in vitro* assembly with the In-Fusion Snap Assembly Master Mix (Takara, 638948).

PW-348, GACTACAAAGACGATGACGACAAGGTGAAGTTGCTGCCAGCCCA

PW-349, CTTGTCGTCATCGTCTTTGTAGTCCATGGTGGGAATTCCAGCACAC

### Co-immunoprecipitation and western blotting

#### Bead preparation:

All the steps were performed using 2-ml low adhesion microcentrifuge tubes (USA Scientific, 1420–2600). A total of 25 μl of magnetic beads per experimental condition were transferred to a tube. The tubes were placed on a magnetic separation rack, and storage buffer was removed. For anti-HA or anti-FLAG magnetic beads, the beads were resuspended in and blocked with 4% BSA in PBS with gentle rocking at RT for 1 hour. For G-protein magnetic beads, the beads were incubated with 3 μl of anti-PCDH15 antibody diluted in 500 μl of Pierce IP Lysis Buffer (Thermo Scientific, 87787) containing protease inhibitors (Thermo Scientific, A32955) for 15 min with gentle rocking at RT. After either process, the tubes were briefly spun to remove the liquid/beads from the cap and placed on a magnetic separation rack for 1 min, and the liquid was removed. The beads were then resuspended in 25-μL Pierce IP Lysis Buffer containing protease inhibitors.

#### Co-Immunoprecipitation:

Single or double plasmid transfections were performed on HEK293T cells 24 hours after passage using FuGENE HD Transfection Reagent (Promega, E2311). Forty-eight hours post-transfection, the cells were rinsed once with 4°C PBS, collected in 1 ml of 4°C PBS using a cell scraper, and then pipetted into an Eppendorf tube. The cells were also pelleted at 4°C at 2,000g for 2 min, resuspended in 300-μL Pierce IP Lysis Buffer containing protease inhibitors, and incubated on ice for 30 min. The resuspended cells were then further disrupted by passing them through a syringe. The lysate was then spun down at 14,000g for 20 min to pellet remaining intact cellular material. A total of 20 μL of lysate was retained for input control on the western blot, and 200-μL lysate was diluted with 300-μL Pierce IP Lysis Buffer containing protease inhibitors. The diluted lysate was added to appropriate beads and incubated with gentle rocking for 1 hour at 4 °C. After incubation, the tubes were spun briefly to remove the beads from the lid and then placed on a magnet rack for 1 min. The lysate was removed, and the samples were washed by resuspending through pipetting in 500-μL lysis buffer. The samples were placed on a magnetic separation rack for 1 min. This wash step was repeated thrice. On the third resuspension, the samples were removed to a new tube.

#### Western Blot:

After the final wash step of co-immunoprecipitation, the tubes were placed on a magnetic separation rack, the buffer was removed, and the samples were resuspended in 40-μL 2x Laemmli Sample Buffer (Bio-Rad, 1610737) containing 0.05% ß-Mercaptoethanol (Bio-Rad, 161–0710). A total of 20-μL 2x Laemmli sample containing 0.05% ß-Mercaptoethanol was added to the input samples, and 20 μL of each sample was run on a Mini-PROTEAN^®^ TGX^™^ Precast Protein Gel, 4%–20% (Bio-Rad, 4561094). The proteins were transferred to the Trans-Blot Turbo Mini 0.2 μm PVDF membrane (Bio-Rad, 1704156) using the Trans-Blot^®^ Turbo^™^ Transfer System (Bio-Rad, 1704150). The blots were blocked overnight at 4 °C with gentle rotation in Intercept^®^ (TBS) Blocking Buffer (LI-COR Biosciences, 927–60001). The blots were incubated with a blocking solution containing 0.1% Tween 20 and primary antibody (1:500 dilution) for 1 hour at RT with gentle rocking. The blots were then washed thrice five min in TBS with 0.1% Tween 20. The blots were then incubated with blocking solution containing 0.1% Tween 20 and secondary antibody (LI-COR Biosciences, 925–32211; 1:5000) for 45 min at RT with gentle rocking in the dark, and they were then washed thrice for 5 min each in TBS with 0.1% Tween 20, with a final rinse in TBS alone. The blots were then exposed using a LI-COR Odyssey CLx system (LI-COR Biosciences).

### Quantification and statistical analysis

Statistical analyses and sample sizes for all the experiments are described in Fig. legends and Table S1. Normality tests were used, and parametric or non-parametric tests were performed as appropriate. Unpaired two-tailed t-tests or the Mann–Whitney test were performed. One-way ANOVA Tukey’s multiple comparisons test was used to compare means across three genotypes. Two-way ANOVA was used to make comparisons across more than two groups followed by multiple test comparisons, either Dunnett’s for comparisons to a reference mean or otherwise Šídák’s. No statistical test was used to pre-determine the sample size; instead, the sample size was determined by animal availability and previous studies in the field. The statistical analysis was performed using GraphPad Prism 9.4 for Mac, GraphPad Software, San Diego, California, USA.

## Data Availability

Any data presented in the paper is available upon reasonable request by contacting N.G.
